# Coaxial Dual-wavelength Interferometric Method for a Thermal Infrared Focal-plane-array with Integrated Gratings

**DOI:** 10.1038/srep25993

**Published:** 2016-05-19

**Authors:** Yuanfang Shang, Xiongying Ye, Liangcai Cao, Pengfei Song, Jinyang Feng

**Affiliations:** 1 Institute of Instrument Science and Technology, Department of Precision Instrument, Tsinghua University, Beijing, 100084, China; 2 Institute of Opto-electronic Engineering, Department of Precision Instrument, Tsinghua University, Beijing, 100084, China

## Abstract

Uncooled infrared (IR) focal-plane-array (FPA) with both large sensing range and high sensitivity is a great challenge due to the limited dynamic range of the detected signals. A coaxial dual-wavelength interferometric system was proposed here to detect thermal-induced displacements of an ultrasensitive FPA based on polyvinyl-chloride(PVC)/gold bimorph cantilevers and carbon nanotube (CNT)-based IR absorbing films. By alternately selecting the two displacement measurements performed by λ_1_ (=640 nm) and λ_2_ (=660 nm), the temperature measuring range with greater than 50% maximum sensitivity can be extended by eight-fold in comparison with the traditional single-wavelength mode. Meanwhile, the relative measurement error over the full measuring range is below 0.4%. In addition, it offers a feasible approach for on-line and on-wafer FPA characterization with great convenience and high efficiency.

Infrared (IR) detection is widely used in various military and civilian applications, such as infrared early-warning, reconnaissance, guidance, night vision and non-invasive medical diagnostics^1^,^2^. Thermal IR imagers, which rely on photo-thermal heating due to IR absorption, have been studied extensively, since they do not require cryogen and have benefits including multi-spectral imaging, low cost and micro volume. For thermal IR imagers, changes in the electric properties^3^,^4^,^5^ or optical properties^6^ of the sensing materials, or thermal-induced deformation of the sensing structures^7^,^8^, induced by the photo-thermal heating effect, can be measured electrically or optically. Commercial thermal IR imagers are almost electrical-readout imagers which have a typical noise-equivalent temperature difference (*NETD*) of 30–40 mK^9^. Compared with the electrical-readout imager, the optical-readout imager benefits from both reduced heat loss and Joule heat noise, indicating an improved IR detection limit, which might be possible to restrain the *NETD* to a range below 5 mK^10^. Since its emergence in 1990s, the optical-readout thermal IR imager has attracted much attention for its potential high resolution. IR focal-plane-arrays (FPAs) based on metal/ceramic bi-material cantilevers have been proposed with various optical IR signal readout methods, including laser beam steering^11^, spatially-filtered optical diffraction^12^,^13^,^14^ and interferometry^15^,^16^,^17^. The laser beam steering technique offers a simple way to measure the deflection with ultrahigh sensitivity. But it is limited to a 1-D array of cantilever-typed structures because crosstalk may appear in a 2-D array. In contrast, the spatially-filtered diffraction and interference techniques based on 4*f* imaging systems are frequently for 2-D thermal IR imagers. However, these techniques are facing a common bottleneck of confined detection range. During the fabrication, the confined measuring range gives rise to high requirements on the cantilever’s initial position and its consistency because the detection sensitivity may be degraded significantly based on the position. Developing thermal IR imagers with both large measuring range and high detection sensitivity is still a big challenge.

Recently, much attention has been poured into the development of FPAs with extended measuring range. Effective optical techniques were proposed to increase the IR signal measuring range while maintaining high detection sensitivity. Gorp *et al.*^18^ and Feng *et al.*^19^ reported an integrated dual-grating method for extended-range interferometric displacement detection, where dual gratings with λ/8 height difference were used to generate a quadrature signal. But this design is inapplicable to FPAs with a small pixel pitch. Another approach to realize constant and high detection sensitivity during a large measuring range is to adjust the sensing structures at the most sensitive point via electrostatic feedback control^20^. This approach is unsuitable for FPAs due to the demand for electrical connections, which will add to the complexity of device and cause adverse heat loss. Ferhanoglu *et al.*^21^ demonstrated a biaxial optical interferometric technique for sensitivity enhancement during a large displacement range. The interference lights were captured either by two CCD cameras after separated with a polarizing beam splitter^22^ or by a single CCD camera through time multiplexing scheme^21^. This method benefits from the advantages of no change in the sensor structure and no need for electrical interconnections at the cost of the complexity of the IR imaging system.

Based on our previous work^23^, in this paper, we propose a novel coaxial 2-λ interferometric method to detect thermal-induced displacements of an ultrasensitive FPA based on polymer-based bimorph cantilevers and CNT films. A single CCD camera is used to capture the 2-λ interference signals without the need for time multiplexing. Absolute position of the FPA could be easily obtained with this coaxial interferometric method. The proposed FPA IR sensor possesses an extended measuring range while maintaining sufficient sensitivity. This coaxial 2-λ interferometric detection method could be extended to different applications of array displacement and position measurement with a high resolution and large range.

## Results

### Thermal IR imager

**Figure 1:**
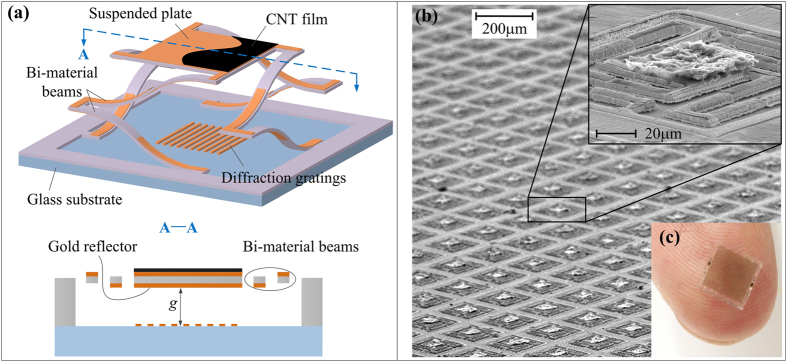
Uncooled infrared focal-plane-array. (**a**) Schematic diagram of the pixel structure and its sectional view. (**b**) SEM images of a fabricated FPA. (**c**) Overall appearance of the fabricated FPA.

In a FPA pixel, a sandwich-structured microplate (gold/PVC/gold) is symmetrically supported by four PVC/gold bi-material microbeams with ultrahigh thermal deformation sensitivity^24^, as is shown in Fig. 1(a). On the front side of the microplate, a carbon nanotube (CNT)-based IR absorbing film exhibits sufficient IR absorption of 90%^25^. On the rear side of the microplate, a gold reflector forms a phase-sensitive grating with a metal grating on the glass substrate. When exposed to IR radiation, the microplate is heated due to the IR absorption and the bi-material microbeams bend in consequence of mismatched thermal stress in the bi-layers. The suspended microplate is thus actuated to move in the vertical direction. Figure 1(b) shows the SEM images of the FPA chip containing 25 × 25 pixels. The size of the suspended microplate is 50 μm × 50 μm and the pixel pitch is 200 μm. For the metal grating on the glass substrate, the period is 7 μm and the width of the gold strips is 3 μm. The gap between the suspended microplate and the substrate is designed to be 2.6 μm. Figure 1(c) shows the overall appearance of the fabricated FPA chip, which measures 4 mm × 4 mm in size. The detailed design of the thermal IR imager can be found in our previous publication^23^.

### Coaxial IR signal readout system

**Figure 2:**
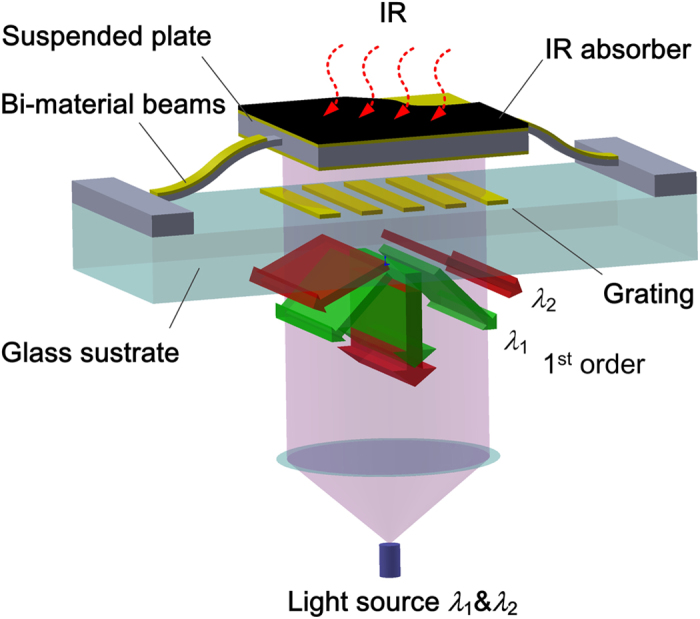
Schematic diagram of a FPA pixel with 2-λ optical readout. The suspended structure absorbs IR radiation and is thermally driven to move vertically. The displacement is monitored by detecting the first diffraction orders for the two wavelengths.

The schematic diagram of the IR signal readout method for a FPA pixel is illustrated in Fig. 2. Two collimated light beams with adjacent wavelength (λ_1_ & λ_2_) illuminate the pixel from the rear side and are diffracted by the grating into different orders at specific angles. For each diffraction order, the interference intensity is modulated by the gap, *g*, between the movable reflector and the metal grating. Under the IR radiation, the thermal-actuated movement of the suspended microplate in the vertical direction changes the interference intensities of the diffracted lights. The IR intensity is thus translated to the optical interference intensities, which are finally measured by a CCD camera.

**Figure 3:**
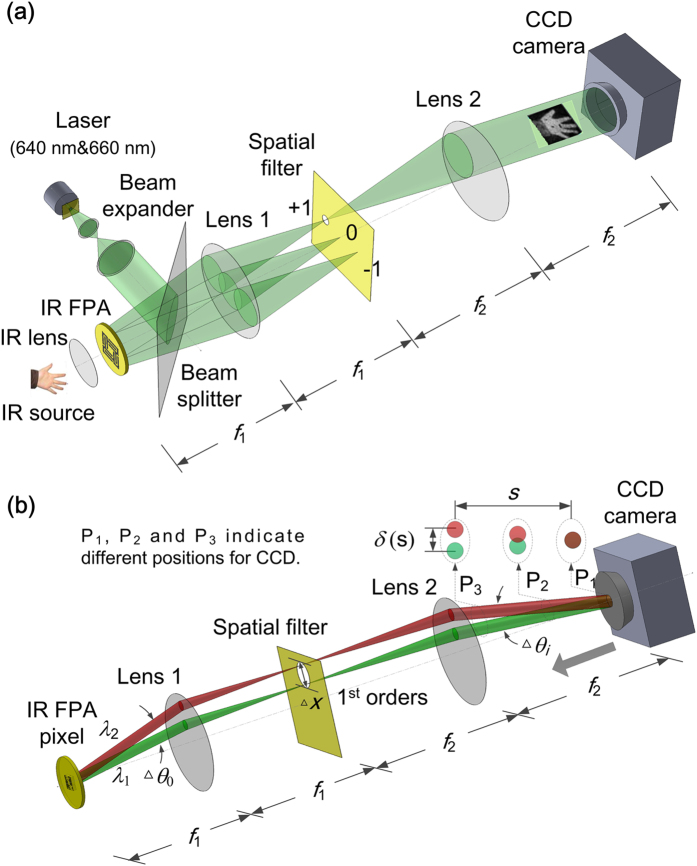
Schematic diagram of (**a**) optical readout system for displacement detection using the first diffraction orders, (**b**) separation of images corresponding to the two wavelengths on a single CCD camera. Lens 1 performs a Fourier transform of the optical signals diffracted from the FPA and lens 2 performs an inverse Fourier transform of the first diffraction orders and reconstructs the FPA’s images on CCD.

Figure 3 schematically illustrates the configuration of the coaxial 2-λ interferometric system for the FPA. As shown in Fig. 3(a), two collimated laser beams (λ_1_ & λ_2_) first pass through a beam expander to illuminate the FPA encapsulated in a temperature-controlled chamber from the rear side. Afterwards, the light beams diffracted by the gratings are focused on the back focal plane by lens 1, where a spatial filter is placed allowing only the first diffraction orders to pass. Finally, images of the FPA are constructed from the first diffraction orders and projected onto a CCD camera by imaging lens 2. As shown in Fig. 3(b), the images produced by the two wavelengths could overlap if CCD is placed on the rear focal plane of lens 2 (indicated by P_1_). To avoid the overlap, CCD is slightly shifted off the focal plane by a certain distance (indicated by P_2_ and P_3_).

**Figure 4:**
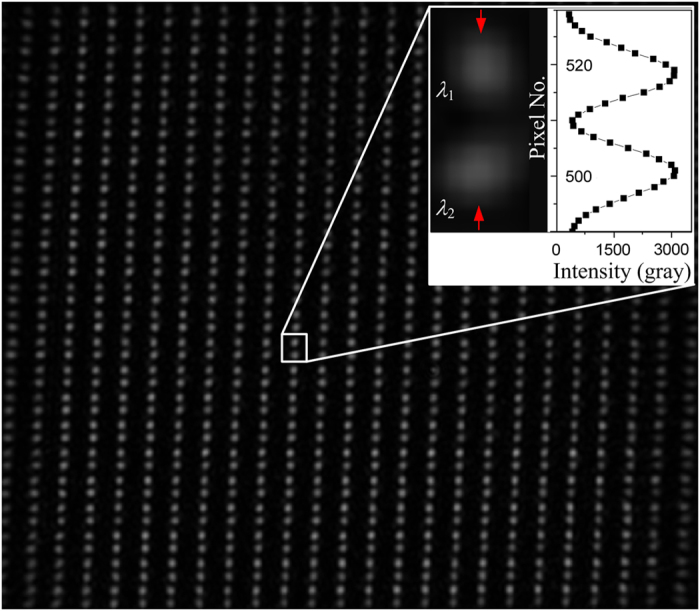
Independent interference light spots corresponding to FPA pixels captured by CCD.

Figure 4 shows an image of the light spots diffracted from the FPA. The light spots resulted from λ_1_ and λ_2_ appear in pairs and correspond to a microplate. It is implied that the size of the movable reflector should be less than half of the pixel pitch so as to completely separate the 2-λ images, which is further validated through the intensity distribution within the spot pairs, as shown in the inset.

### Temperature sensing

**Figure 5:**
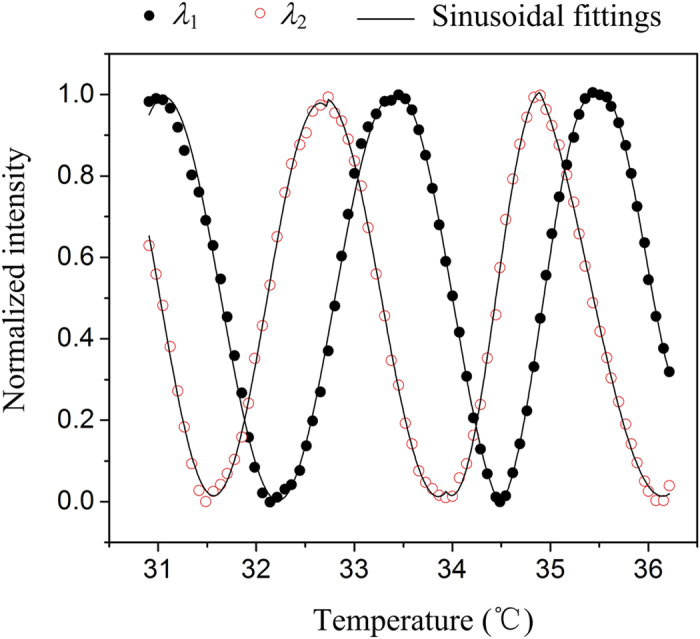
Light intensity response of the fabricated FPA measured with 2-*λ* interferometric method to temperature.

The thermal IR imager is essentially sensitive to temperature variation. To give a primary assess of the fabricated FPA, the temperature sensing performance is characterized by heating the FPA from 30.9 °C to 36.2 °C and monitoring the interference light intensities with the 2-λ readout system. Figure 5 shows the measured normalized intensity of a pair of light spots for λ_1_ and λ_2_ versus temperature. Considering the fact that the thermal deformation of the PVC/gold microbeams (and thus the gap between the suspended microplates and the substrate) changes linearly with temperature^19^, the curves can be regarded as sinusoidal function. By dividing the the normalized intensity-temperature signals into sequential sections according to the peaks and valleys and fitting them with sinusoidal function individually (to reduce the fitting error over the full measuring range), we have


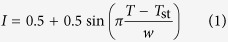


where *T* and *T*_st_ are the FPA’s temperatures within and at the starting point of a certain section, respectively. *w* is half-period of the intensity-temperature curve in this section. As shown in Fig. 5, the curves coincide quite well with sinusoidal function. The fitting correlation coefficients are above 99.7% in all sections. *w* was fitted to be 1.1 ± 0.1 K, revealing a thermal deformation sensitivity *S*_td_ (=λ/4*w*, thermal displacement per temperature rise) of 143 ± 18 nm/K for the FPA during this temperature range.

### Absolute position measurement

Furthermore, the absolute gap between the suspended microplate and the substrate can be determined from the 2-λ interference signals^20^. Within the displacement measuring range (λ_s_/4 ~ 5.28 μm), the gap, *g*, can be obtained from the currently-acquired normalized intensity *I*_N_ and the previously-acquired normalized intensity *I*_N_’, according to:


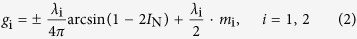


where *m*_1_ is an integer between 0 and 33 and *m*_2_ (=*m*_1_ or *m*_1_ − 1) is an integer between 0 and 32. The first expression takes positive sign when arcsin(1 − 2*I*_N_) > arcsin(1 − 2*I*_N_’), while takes negative sign when arcsin(1 − 2*I*_N_) < arcsin(1 − 2*I*_N_’). *g*_1_ and *g*_2_ determined from *m*_1_ and *m*_2_ when |*g*_1_ − *g*_2_| is minimized represents the absolute gap. Based on Eq.(2), the absolute gap under 31 °C was estimated to be 2.63 μm, which is close to the designed 2.6 μm. The results significantly verify the feasibility of absolute position measurement with our proposed coaxial 2-λ interference method.

With the absolute position measurement ability of the coaxial 2-λ readout system, the absolute temperature *T* can be detected by using a controlled reference temperature *T*_ref_ at the initial gap *g*_0_.


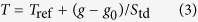


where *g* is the measured absolute gap of the suspended microplate and the substrate and *S*_td_ is the thermal deformation sensitivity of the thermal sensing structures in the FPAs. In addition, the manufacturing quality can be evaluated by measuring the absolute gap of the fabricated FPA.

## Discussion

Developing IR imagers with both large measuring range and high detection sensitivity is still a challenge since the dynamic range of the detected signals is very limited in the traditional measuring methods. For the paramount uncooled IR FPAs, the optical IR signal readout technique is of great importance, because it offers distinctive advantages such as wireless measurement, low noise, negligible heat loss and scalability through existing MEMS processing technology. Unfortunately, few optical techniques meet the demand of both large measuring range and high detection sensitivity. In addition, most optical techniques exhibit high requirements on manufacturing quality of the FPA, thus seriously reducing the yield. In this work, a polymer-based bimorph type FPA was combined with a coaxial 2-λ optical interferometric system based on diffraction gratings to read the IR signal with high sensitivity by measuring the thermal-induced displacements. The IR signals detected by the FPA can be figured out from the measured interference intensities. And the phase-shifted feature of the 2-λ interference signals could be utilized to extend the measuring range by switching between them to select signals with larger slope (higher detection sensitivity).

**Figure 6:**
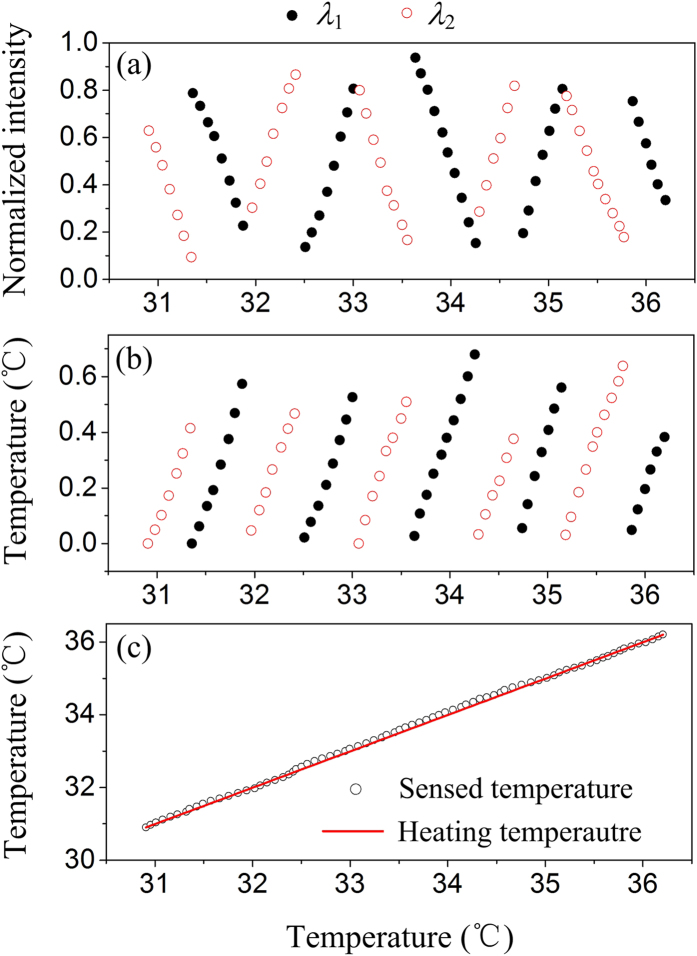
Temperature sensing of the FPA tested with the 2-λ readout method. (**a**) Normalized intensity sections alternately selected with large slopes. (**b**) Resolved sensed temperature from the selected signal sections in (**a**). (**c**) Spliced temperature variations from (**b**) over the full measuring range. The solid and hollow circle symbols in (**a**,**b**) represent the data for λ_1_ (=640 nm) and λ_2_ (=660 nm), respectively. The hollow circle symbol and solid line in (**c**) represent for sensed and heating temperatures, respectively.

**Figure 7:**
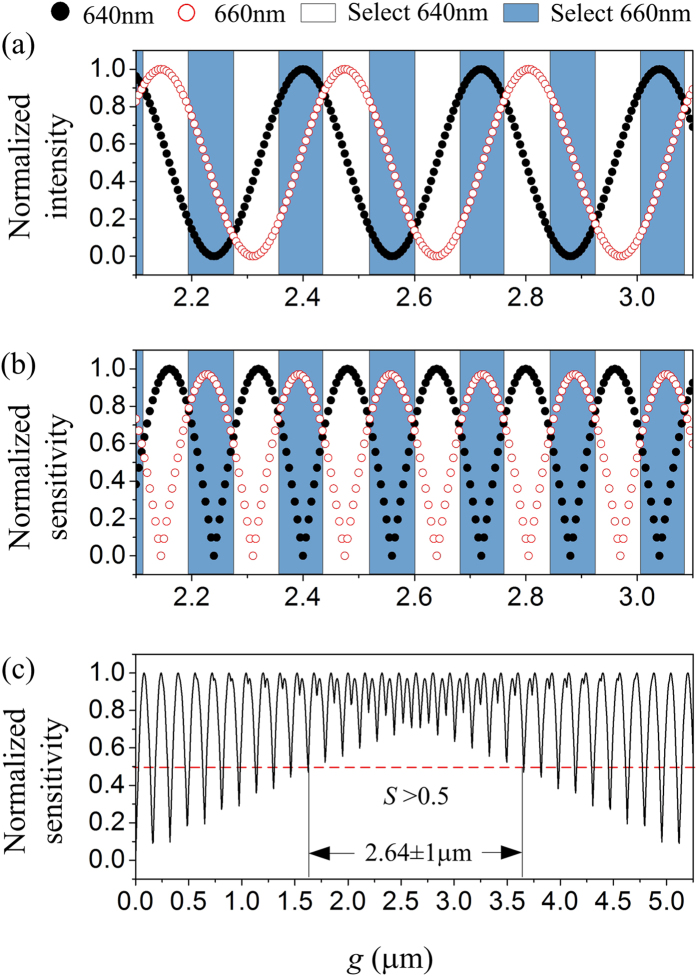
Simulation of 2-*λ* interference detection. (**a**,**b**) are normalized interference light intensity and optical detection sensitivity for the two wavelengths, respectively. (**c**) Combined sensitivity. The solid and hollow symbols in (**a**,**b**) represent for λ_1_ (=640 nm) and λ_2_ (=660 nm), respectively. The rectangle boxes represent the alternately selecting of λ_1_ and λ_2_ interference signals. The dashed-line in (**c**) represents for 50% maximum sensitivity.

Figure 6(a) shows the alternately selected multi-sectional signals according to the 2-λ switching scheme described in the section of methods. The measured temperature variations in these selected signal sections can be resolved with Eq. (4), as plotted in Fig. 6(b),


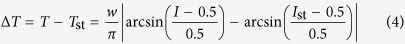


where *I* and *I*_st_ are the normalized intensities within and at the starting point of a certain selected section in Fig. 6(a), respectively. As shown in Fig. 6(c), by splicing the resolved temperature variation from all the selected sections, the maximum detectable temperature range was significantly extended. The absolute temperature of the sensing structure (and thus that of the IR source) was figured out with the original temperature of the heating chamber as a reference. For comparison, the heating temperature of the chamber is also plotted. It is clearly seen that the sensed temperature by the FPA shows good agreement with the heating temperature of the chamber. With the presented coaxial 2-λ interference method, temperature variation as large as 5.3 °C was successfully detected by the FPA with a constant sensitivity and a relative error below 0.4% in the full range. The corresponding thermal displacement measured was as large as 800 nm, which was extended by a factor of eight compared to the single-wavelength interference method (λ_1_ = 640 nm). As discussed in the section of methods, displacement measurement up to 2 μm is feasible for the presented method, according to Fig. 7(c) in the section of method. Thus, the FPA should be capable of measuring IR-induced temperature up to 13.3 °C, which is sufficient for most IR detection applications.

As discussed above, the IR signal readout system based on the coaxial 2-λ interference technique presented in this paper is able to obviously extend the measuring range while maintaining a high and constant sensitivity. The coaxial optical system needs no change in the sensor structure and requires neither a double-camera configuration nor a time multiplexing system, thus simplifying the optics implementation and leading to a more compact IR imaging system. The proposed system is of great convenience and high efficiency due to the direct imaging and simultaneous IR signal readout of a 2-D structure array. Meanwhile, it promises an easy technique to evaluate the devices’ manufacturing quality. Therefore, the optical IR signal readout method presented here is simple, low-cost, easy-to-use and adaptable.

In addition, the temperature resolution (*NETD*) and the spatial resolution are another two characteristics of great importance for IR imagers. However, when the pixel pitch is reduced to improve the spatial resolution, the FPA’s *NETD* will be deteriorated as well, due to the decrease of the IR absorption area and the length of the bi-material microbeams. Fortunately, for FPAs with smaller pitch and larger format (and thus higher spatial resolution), the *NETD* can be significantly enhanced by structure optimization, including designing narrower beams and gaps between them^26^, or constructing double-layer thermal sensing structures, demonstrated by Wang *et al.* in^27^. Besides, increasing the displacement resolution of the optical readout system can improve the FPA’s *NETD* directly. To meet the demand for capturing the images of the massive FPA pixels with small pitch, an optical readout system should be implemented with a large-format and small-pitch CCD to provide the required spatial resolution.

As an example, we further designed a highly integrated FPA with a smaller pixel pitch of 30 μm to improve the spatial resolution, while retain superior *NETD*. The design parameters are listed in Table 1. By reducing the beam width *w*_beam_ and space *w*_space_ of the 2-folded microbeams to ~1 μm and ~0.5 μm, respectively, the length of the bi-material microbeams *L*_FOB_ was extended from 70% to 1.47 times of the pixel pitch and the efficient IR absorption area was increased from 6.3% to 68.6% of the pixel area. The length of the thermal isolation legs, *L*_iso_, between the suspended structures and the substrate was optimized to 5 μm to realize a large heat transfer efficiency *H* (ratio of the temperature rise of the FPA to that of the IR object) ~ 0.015 and a short response time τ ~53 ms. The thickness of PVC layer was reduced to ~0.3 μm and that of Au layer was ~40 nm. By designing narrower and thinner beams to increase the deformation length and enhance the thermomechanical response, *S*_td_ was simulated to be ~156.2 nm/K.

In our optical configuration, the metal gratings on the substrate were designed to be 7 periods with a periodicity of 2 μm, whose size is smaller than half of the pixel pitch to ensure the spots of 2-λ light to fall onto a pixel area in the CCD image plate and the number of periods is larger than four to ensure the displacement detection accuracy^28^. On the other hand, as indicated by the slope of the curves in Fig. 6(c), the optical readout system responses to the heating temperature of the FPA with a sensitivity *S*_to_ ~ 1.13 K^−1^. Considering *S*_td_ ~ 143 nm/K for the FPA with 200 μm pixel pitch, the sensitivity of the optical displacement detection *S*_do_ (*=S*_to_/*S*_td_) was revealed to be ~ 0.008 nm^−1^. Assuming that the optical readout noise *N*_noise,op_ was suppressed to ~ 1 gray by adopting a temperature-controlled 12-bit camera, an ultrastable dual-wavelength laser and an appropriate digital filtering algorithm, the resolution of the optical displacement detection was estimated to ~ 0.03 nm (=*N*_noise,op_/2^12^/*S*_do_), which was manifested by the literature^29^ that reports the resolution for the interferometric displacement detection method based on integrated diffraction reaching to sub Angstrom resolution (∼0.02 Å). The *NETD*_op_ originated from the optical readout system was estimated to be 12.8 mK (=0.03 nm/*H*/*S*_td_). For the temperature control accuracy *N*_noise,tf_ ~50 μk of the camera, *NETD*_tf_ originated from the surrounding temperature fluctuation was estimated to be 3.3 mK (=*N*_noise,tf_/*H*). So, the thermal IR imager with coaxial dual-wavelength interferometric system and 30 μm-pitch FPA has the potential to realize a *NETD* of about 13.2 mK, basically meeting the demands for IR imaging.

## Methods

### Optical interferometric displacement detection

The interference intensity of the first diffraction order *I*_±1_ and the displacement detection sensitivity *S*_di_ can be expressed as:


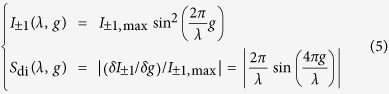


where *I*_±1,max_ is the peak intensity of the first diffraction order and λ is the wavelength of the collimated light. As seen from Eq. (5), *S*_di_ varies sinusoidally with respect to *g*. The maximum sensitivity is obtained at *g* = (2*k* + 1)λ/8 and the minimum sensitivity at *g* = *k*λ/4, where *k* is a non-negative integer. It is found out that the single-wavelength displacement detection with greater than 50% maximum sensitivity is limited to an unambiguous range of λ/6.

By simultaneously monitoring the first diffraction orders corresponding to the two wavelengths, we can alternately select the optical intensity signal with higher detection sensitivity to avoid insensitive ranges. The so-called combined sensitivity *S*_di,2λ_ and beat wavelength λ_s_ for the 2-λ readout signal can be described as:


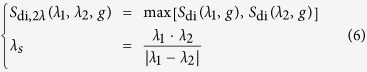


According to Eqs (5) and (6), the maximum sensitivity of the 2-λ displacement detection occurs at *g* = (2*k* + 1) λ_s_/8. Figure 6 shows the simulation results of a 2-λ displacement readout, where λ_1_ = 640 nm and λ_2_ = 660 nm. As shown in Fig. 7(a), the optical intensity signals are normalized and divided into sequential sections according to their detection sensitivities. In the color-filled sections, the detection sensitivity for λ_2_ is larger than that for λ_1_, while in the adjacent unfilled sections, the relation reverses, shown in Fig. 7(b). In each section, the optical signal with higher detection sensitivity is selected, leading to a combined sensitivity plotted in Fig. 7(c). The combined sensitivity reaches the maximum value at *g* = 2.64 μm (~ λ_s_/8), displaying a measuring range of 2 μm with greater than 50% maximum sensitivity. The horizontal axis in Fig. 7 represents for the gap between the suspended plate and the substrate.

### Optical readout system configuration

The FPA is heated by the temperature-controlled chamber with a resolution of 0.01 °C and the modulated intensities are monitored by a 12-bit CCD camera (CoolSNAP EZ, bought from Photometrics, USA). A dual-wavelength laser module (bought from Haoliang Photoelectric Equipment Co., Ltd., China) with λ_1_ = 640 nm and λ_2_ = 660 nm is adopted. The focal lengths of lens 1 and lens 2 are 180 mm and 250 mm, respectively. To reduce the random-fluctuation noise, intensities of 5 × 5 CCD pixels within each light spot is averaged to provide the output interference intensity.

The offset distance (indicated by P_1_, P_2_, P_3_ in Fig. 3(b)) is determined by the diffraction angles of the two wavelengths, the size of the microplates and the focal lengths of the optical lens. Based on the theory of diffraction gratings, the diffraction angle for the first-order diffracted light is given as:


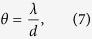


where λ is the wavelength and *d* = 7 μm is the grating period. Due to the difference of the diffraction angles Δ*θ*_o_ = (λ_2_ − λ_1_)/*d*, the focused light spots for the two wavelengths are separated by a distance Δ*x* on the filter plane, as:


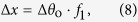


where *f*_1_ and *f*_2_ are the focal lengths of lens 1 and lens 2, respectively.

The image size of the microplate on the CCD is given as:


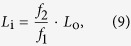


where *L*_0_ is the size of the microplate (the actual size).

For a certain offset distance, *s*, of the CCD, the 2-λ images is separated by a distance δ(*s*), which is estimated as:


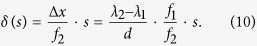


Here, δ(*s*) must be larger than *L*_i_ to completely separate the 2-λ images. In our work, δ(*s*) is set to be 2*L*_i_. Consequently, *s* is estimated to be 67 mm, according to Eqs (7)–(10).

## Additional Information

**How to cite this article**: Shang, Y. *et al.* Coaxial Dual-wavelength Interferometric Method for a Thermal Infrared Focal-plane-array with Integrated Gratings. *Sci. Rep.* **6**, 25993; doi: 10.1038/srep25993 (2016).

## Figures and Tables

**Table 1:** Parameters for the redesigned FPA.

Pixel pitch (μm)	*N*	*w*_beam_ (μm)	*w*_space_ (μm)	*L*_iso_ (μm)	*L*_FOB_ (μm)	Thickness of PVC (μm)	Thickness of Au (nm)	Fill factor
30	2	1	0.5	5	44	0.3	40	68.6%
*H*	*S*_td_ (nm/K)	τ (ms)	*NETD*_tf_ (mK)	*NETD*_op_ (mK)	*NETD* (mK)			
0.015	156.2	53	3.3	12.8	13.2			
